# Machine Learning Algorithm to Predict Acidemia Using Electronic Fetal Monitoring Recording Parameters

**DOI:** 10.3390/e24010068

**Published:** 2021-12-30

**Authors:** Javier Esteban-Escaño, Berta Castán, Sergio Castán, Marta Chóliz-Ezquerro, César Asensio, Antonio R. Laliena, Gerardo Sanz-Enguita, Gerardo Sanz, Luis Mariano Esteban, Ricardo Savirón

**Affiliations:** 1 Department of Electronic Engineering and Communications, Escuela Universitaria Politécnica de La Almunia, Universidad de Zaragoza, Calle Mayor 5, 50100 La Almunia de Doña Godina, Spain; javeste@unizar.es; 2 Department of Obstetrics and Gynecology, San Pedro Hospital, Calle Piqueras 98, 26006 Logroño, Spain; bcastan@riojasalud.es; 3 Department of Obstetrics and Gynecology, Miguel Servet University Hospital, Paseo Isabel La Católica 3, 50009 Zaragoza, Spain; 4 Department of Obstetrics, Dexeus University Hospital, Gran Via de Carles III 71-75, 08028 Barcelona, Spain; martacholiz@gmail.com; 5 Department of Applied Mathematics, Escuela Universitaria Politécnica de La Almunia, Universidad de Zaragoza, Calle Mayor 5, 50100 La Almunia de Doña Godina, Spain; casencha@unizar.es (C.A.); arlalibi@unizar.es (A.R.L.); 6 Department of Applied Physics, Escuela Universitaria Politécnica de La Almunia, Universidad de Zaragoza, Calle Mayor 5, 50100 La Almunia de Doña Godina, Spain; cherraldin@unizar.es; 7 Department of Statistical Methods and Institute for Biocomputation and Physics of Complex Systems-BIFI, University of Zaragoza, Calle Pedro Cerbuna 12, 50009 Zaragoza, Spain; gerardo@unizar.es; 8 Department of Obstetrics and Gynecology, Hospital Clínico San Carlos and Instituto de Investigación Sanitaria San Carlos (IdISSC), Universidad Complutense, Calle del Prof Martín Lagos s/n, 28040 Madrid, Spain; rsaviron@gmail.com

**Keywords:** electronic fetal monitoring, fetal heart rate, sensors, acidemia, machine learning, random forest, clinical utility curve

## Abstract

Background: Electronic fetal monitoring (EFM) is the universal method for the surveillance of fetal well-being in intrapartum. Our objective was to predict acidemia from fetal heart signal features using machine learning algorithms. Methods: A case–control 1:2 study was carried out compromising 378 infants, born in the Miguel Servet University Hospital, Spain. Neonatal acidemia was defined as pH < 7.10. Using EFM recording logistic regression, random forest and neural networks models were built to predict acidemia. Validation of models was performed by means of discrimination, calibration, and clinical utility. Results: Best performance was attained using a random forest model built with 100 trees. The discrimination ability was good, with an area under the Receiver Operating Characteristic curve (AUC) of 0.865. The calibration showed a slight overestimation of acidemia occurrence for probabilities above 0.4. The clinical utility showed that for 33% cutoff point, missing 5% of acidotic cases, 46% of unnecessary cesarean sections could be prevented. Logistic regression and neural networks showed similar discrimination ability but with worse calibration and clinical utility. Conclusions: The combination of the variables extracted from EFM recording provided a predictive model of acidemia that showed good accuracy and provides a practical tool to prevent unnecessary cesarean sections.

## 1. Introduction

Currently, the universal method for the surveillance of intrapartum fetal well-being is the continuous monitoring of fetal heart rate (FHR) and maternal uterine contraction (UC) signals [1]. Electronic fetal monitoring (EFM) requires complex electronic devices developed to acquire, process, and display the signal. In the intrapartum period, an ultrasound transducer is used for the external FHR monitoring. This transducer contains piezoelectric effect crystals that convert electrical energy into ultrasound waves and uses the Doppler effect to detect movements of the cardiac structures [2,3]. In this context, several systems have been developed for central monitoring of fetal signals to provide simultaneous display of multiple tracings on several locations, allowing easier monitoring of signals [4]. The rate and pattern of the fetal heart are displayed on the computer screen and printed onto special graph paper.

Shannon defined entropy as a measure of the average information provided by a set of events and informs on its uncertainty [5]. The information theory is a mathematical theory of communication to quantify information. Information theory has been successfully used to evaluate biological biochemical signal networks [6] or in evolutionary biology [7]. Metrics such as mutual information have been used in the information theory in order to quantify the sharing of information in the presence of anomalies in electrocardiographic heart signals [8]. Fetal heart rate is altered in the presence of adverse fetal problems, the level of chaoticity in the signal may be measured using entropy. Higher entropy represents higher uncertainty and a more irregular behavior of the signal. Entropy can even explain how linked complex systems interact and exchange information.

The prediction of acidemia understood as fetal asphyxia was mainly based on the visualization of morphological aspects of fetal heart recording (FHR) with limited accuracy [9]. The quantification of the magnitude of this information becomes a goal in the study of FHR signals. Guidelines, such as the American College of Obstetricians and Gynecologists (ACOG) [10,11], proposed the categorization of FHR parameters to predict acidemia, but most categorization systems show lack of accuracy [12]. In addition, the interobserver agreement between experts shows the need to make the prediction of acidosis through the modeling of the EFM characteristics rather than the visual interpretation of the signal [13].

Two main objectives focused the effort on the improvement of the diagnosis of acidemia in recent years, the proposal of new predictors derived from the fetal cardiotocography (CTG) and their combination with previous features [14,15]. Automated systems can extract data on the FHR [16] or patterns can be obtained using signal processing as fractal analysis [17,18], but regarding combination of EFM variables, the artificial intelligence and machine learning algorithms have opened a range of possible applications with multiple development [19,20,21,22].

Machine learning algorithms had helped to improve prediction in different problems in medicine [23], although the nature of the used models is very diverse. Decision trees [24], support vector machines [24,25,26], adaptative boosting [24], convolutional neural networks [27,28], neuro fuzzy inference systems [29], neural networks [25,29], deep stacked sparse auto-encoders [29], or deep-ANFIS models [29] are machine learning techniques used for acidemia prediction. Machine learning algorithms are based on the minimization of a loss function. The cross-entropy is a generalized loss function that can be interpreted as an information measure [30], best models correspond to the minimum discrimination information [31]. Abnormalities in the FHR tend to increase the cross-entropy function, showing it as a candidate for quantifying the variety of physiological signals.

The success of machine learning models was distributed in a wide range, and can be classified in two groups, models that were built from the FHR signal and others built with the variables extracted from the signal. The most frequent parameters used to validate these previous models were the area under the receiver operating characteristic (ROC) curve [32], or the sensitivity and specificity that corresponds to a threshold probability of acidemia. To our knowledge, none, or very few of the developed machine learning models analyzed the clinical utility of these models although this is one the most important properties for the applicability of a prediction model [33].

Complementing the prediction of acidosis [34,35,36], recent publications have analyzed the importance of deceleration physiology and use parameters such as the deceleration area, that reports accumulated hypoxemia [14,37]. In addition, it is essential to know about the fetal time available to recover between deceleration and fetal ability to repeatedly activate the chemoreflex, fetal resilience [38,39]. Moreover, combining these parameters can provide better-adjusted predictions, the fetal reserve index is a promising classification system that proposed the improvement of EFM by adding three clinical variables: maternal, obstetrical, and fetal risk-related information in a scoring system to assess fetal perfusion and resilience rather than “hypoxia” [40].

In a previous study, we analyze a new parameter, the total reperfusion time (fetal resilience) to predict fetal acidemia [15]. In this study, we build a predictive model of acidemia using the FHR variables extracted from the EFM recording, including the reperfusion time, in a case–control study. For the combination of variables, we used the multivariate logistic regression, random forest, and neural networks models, performing a complete validation based on the analysis of the discrimination, calibration, and clinical utility of models.

## 2. Materials and Methods

### 2.1. Study Design and Patients Recruitment

The study was designed as a retrospective case–control analysis that involves pregnancy data recruited between June 2017 and October 2018 at the Miguel Servet University Hospital, in Zaragoza, Spain. The inclusion criteria were singleton term gestation between 37 and 42 weeks, cephalic presentation, and no fetal anomalies. In addition, we selected electrocardiographic recordings showing presence of a deceleration pattern in the EFM defined as two or more decelerations in the last 30 min. As exclusion criteria, we defined having experienced a sentinel event (uterine rupture, cord prolapse, or shoulder dystocia), EFM with less than 30 min registered period, or anomalies that do not enable the analysis of EFM. In the case of a monitoring that had not started active labor, the EFM register was discarded.

**Figure 1:**
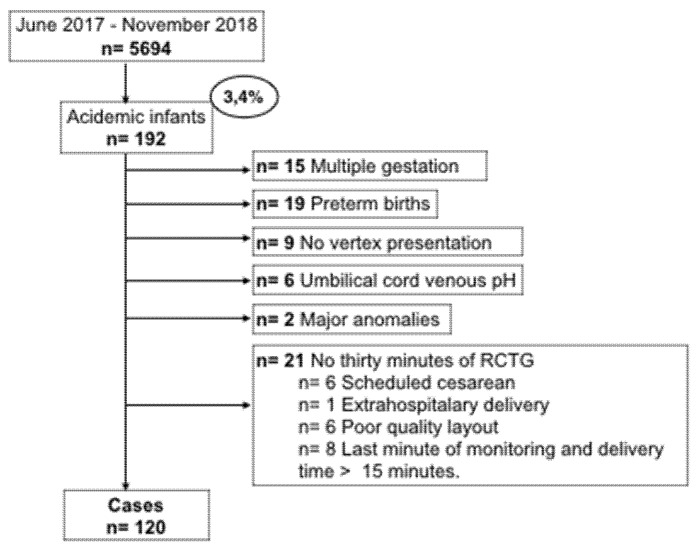
Flow chart of patient recruitment.

The outcome of the study was neonatal acidemia defined as pH < 7.10, measured by arterial cord blood at birth, these are the cases of the analysis. From the 5694 women in the initial cohort, 192 (3.4%) infants were acidotic. In Figure 1 we show the flowchart of the study, 72 acidemic fetuses were excluded from the analysis for lack of criteria. The remaining 120 infants with arterial acidemia were included as cases, together with 258 in the control group. The controls were selected using a non-randomized 1:2 consecutive type method; each selected control is chronologically consecutive to a case, selecting two controls for each case.

We additionally recruited maternal and pregnancy information on parity, maternal age, maternal pathologies, gestational age at birth, birthweight, estimated percentile weight, and fetal gender.

### 2.2. Electronic Fetal Monitoring

**Figure 2:**
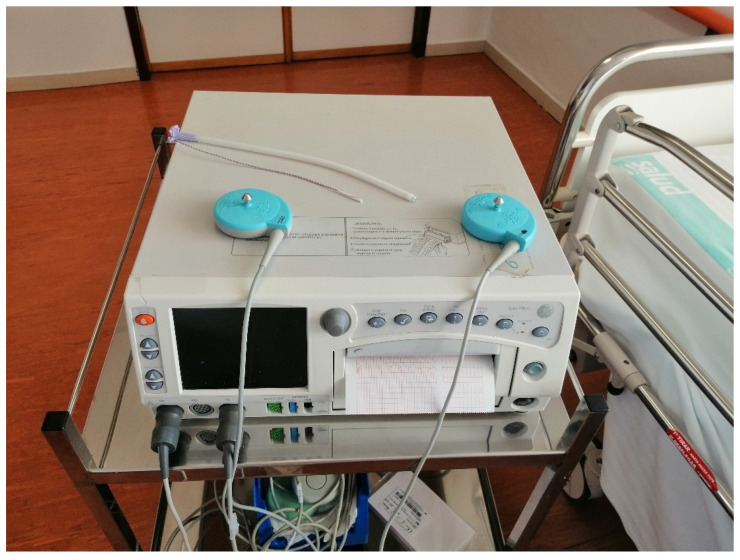
Electronic fetal monitoring.

For the monitoring of fetal well-being, as can be seen in Figure 2, a fetal activity supervisor Corometrix 256CX was used. Two sensors were employed for this task: an ultrasonic transducer to capture the electrocardiographic (ECG) fetal activity and a TOCO (Tocotonometer) transducer to capture the uterine activity. Both were attached to the mother with binding bands and the coming signals were analyzed continuously by obstetricians during the final process of pregnancy prior to delivery.

The ultrasound transducer is placed on the maternal abdomen by means of one belt and transmits the ultrasonic signal of the fetal heart. It operates with a pulse repetition frequency of 4 kHz, a pulse duration of 92 uS, and a transmission frequency of 1151 MHz. It is capable of measuring heart rate from 50 to 210 bpm and its precision is 1 bpm.

The TOCO transducer is also placed on the maternal abdomen by means of one belt and it detects the forward displacement of the maternal abdominal muscles during a contraction. The TOCO transducer is composed of several strain gauges configurated to transduce pressure measurements into displacement. This device can measure pressures from 0 to 13.3 KPa with a resolution of 0.13 kPa and a bandwidth from 0 to 0.5 Hz.

In our study, the last 30 min of EFM prior to delivery were retrospectively analyzed and interpreted between two obstetricians attached to the delivery section, blind to the neonatal outcome, using the criteria and the patterns described in the Category system of the Eunice Kennedy Shriver National Institute of Child Health and Human Development (NICHD) [41]. Five elements of the EFM recording were extracted using the definitions from the NICHD criteria and then used to categorize the EFM recordings into one of the three accepted categories: Category I, Category II, or Category III to describe EFM data.

**Figure 3:**
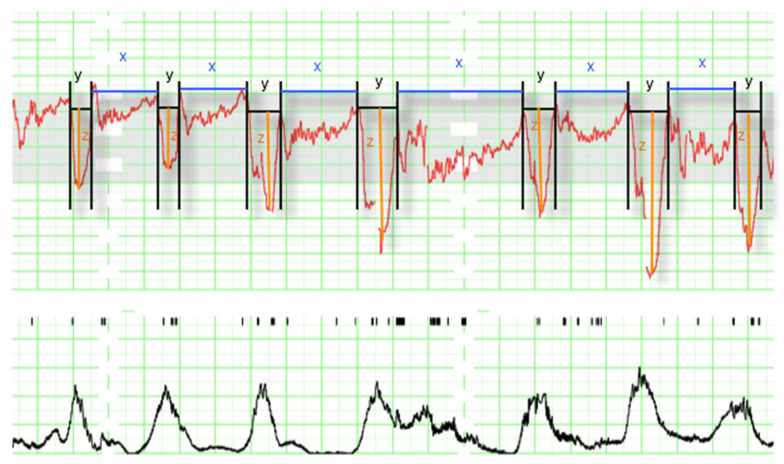
Intrapartum electronic fetal monitoring analysis (1 cm/min). The panel contains the fetus signal (**above**), where the following parameters can be observed: decelerations (y), time of reperfusion (x), and depth of deceleration (z); and the mother’s uterine contractions measured in mm Hg (**below**) not used for the analysis.

Additionally, as our purpose was to use machine learning algorithms in order to predict acidemia, we recruited information about the non-NICHD parameters These parameters were obtained from the EFM recordings as it is described in Figure 3. In the graph it can be seen the electrocardiographic fetus signal measured in beats per minute (above) and the mother’s uterine contractions measured as mm Hg (below).

We divided the EFM signal into deceleration (y) and interdeceleration (x) periods. The duration of reperfusions was defined as x (interdeceleration time), the duration of decelerations was defined as y (deceleration time), and the depth of decelerations as z.

From x, y, and z, we calculated the parameters:

- Total reperfusion time as the sum in minutes of the period that the fetus remains at baseline without deceleration during the last 30 min ∑x.
- Deceleration time as the sum in minutes of the period of time that the fetus is decelerating during the last 30 min ∑y.
- Total deceleration area as the sum of all areas of deceleration, being the deceleration area the product of the duration of deceleration in seconds and its maximum depth of fall from baseline expressed in beats per minute divided by two $\sum \frac{yz}{2}$.

Additionally, we considered for the multivariate model the following variables: number of decelerations, minimum beats per minute (bpm), number of decelerations greater than 60 s, number of decelerations greater than 60 beats per minute in depth, and the presence of decelerations in more than 50% of contractions, considered to be recurring, thus we defined the variables that describe the occurrence of recurrent decelerations greater than 60 s, and recurrent decelerations with depth > 60 bpm.

### 2.3. Statistical Analysis

We descriptively analyzed data comparing acidotic and non-acidotic infants. The continuous variables were summarized by median and interquartile range (IQ) and categorical variables by absolute and relative frequency of each category. Differences between acidotic and non-acidotic groups were analyzed using the Mann Whitney or Chi-square test for continuous or categorical data.

To predict acidotic infants in the last 30 min of labor, multivariate models were built using logistic regression models, random forest, and neural networks. For building and testing models the original database was randomly split into training (80%) and validation data (20%).

Validation of models was estimated by its discrimination measured by means of the area under the receiver characteristic curve (AUC), and its calibration through calibration curves and of the two informative parameters: ‘intercept’ (calibration-in-the-large) that measures the difference between average predictions and average outcome; and ‘slope’, which reflects the average effect of predictions on the outcome [42]. The AUC can be interpreted as the probability that the model assigns a greater probability of being acidotic for an acidotic case rather than a non-acidotic case, it ranges from 0 to 1, corresponding the 0.5 value to a random model, 0.7 to an acceptable model, 0.8 to a good model, 0.9 excellent model, and 1 perfect discrimination. The 95% confidence intervals for AUC were calculated using DeLong estimation [43]. The calibration curve analyzes graphically the concordance between predictions and the real occurrence of the outcome, a perfect calibration corresponds with the diagonal line. The predictive ability of the models summarized by their AUC was compared using the De Long test [43].

We also analyzed the clinical utility of the developed machine learning models. This property analyzes the practical use of a prediction model, that as a dichotomic classification model, using a cutoff point that classified individuals as positive (1) or negative (0), above or below the cutoff point. Several methods have been implemented for this purpose, probably the most used is the decision curve [44], that measures for different cutoff points the net benefit of the application of the model in comparison to classify all individual as 0 or 1, that also can be applied to compare models. Although this proposal provides a good guide to select the range of cutoff points with good net benefit, their interpretation is a weighted estimation and cannot be interpreted as a parameter with an easy clinical interpretation. Predictiveness curve also analyze the benefit of the application of a model, but with a less wide diffusion in this field [45].

Here, we used to analyze the clinical utility of the developed models the clinical utility curve [46] that we proposed previously in prostate cancer prediction with satisfactory results. In this curve, the X axis corresponds to the threshold probability to consider a neonate as acidotic, and on the Y axis we represent the percentage for two different measures. The first corresponds to the percentage of missing acidotic infants below the selected cut-off point, and the second one to the number of infants below the cut-off point. Using this curve for different cutoff points we can evaluate the percentage of acidotic fetuses with a wrong classification, and the fetuses with a very low risk of acidemia that are going to be saved from an unnecessary cesarean section for loss of fetal well-being, that are clinical practice parameters.

All analyses were performed using the R language programming v.4.0.3 (The R foundation for statistical computing, Vienna, Austria) with the addition of the rms, randomForestSRC, nnet, neuralnet, and NeuralNetTools libraries [47].

## 3. Results

### 3.1. Descriptive Analysis

Descriptive analysis of data is shown in Table 1. In the maternal–fetal variables of the study, we found statistically significant differences between acidotic and non-acidotic groups in the nulliparity, type of delivery, and SGA variables. Regarding EFM variables, the ACOG categories, № Decelerations > 60 sg, Recurrent decelerations > 60 sg, № Decelerations depth > 60 bpm, Recurrent decelerations depth > 60 bpm, Deceleration area, Minimum deep bpm, Maximum deep bpm, and Mean deep bpm showed differences between groups.

### 3.2. Multivariable Prediction of Acidemia

#### 3.2.1. Building Models

To predict acidemia we used a traditional approach in classification problems as the logistic regression model, and the machine learning algorithms: random forest and neural networks.

The logistic regression model was built using a backward stepwise selection process. In Table 2 we show the significant variables in the multivariate analysis.

The model showed good accuracy, with an AUC value in training data (80% data) of 0.826 (0.778–0.875) (95% confidence interval (C.I.)), and 0.840 (0.750–0.930 95% C.I.) in validation data (20% data).

Regarding the additive model of classification trees that is the random forest, it was training with different set of parameters, and the best model was attained with the configuration shown in Table 3.

**Figure 4:**
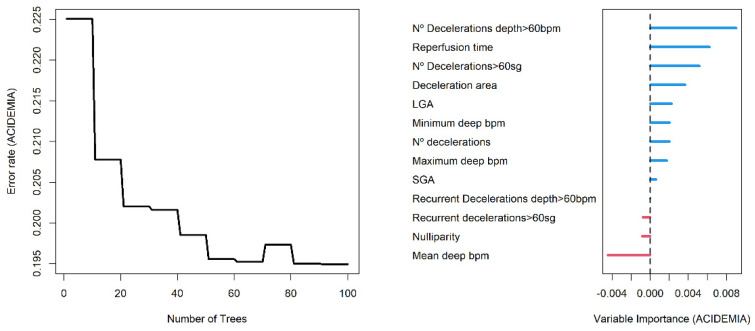
Error rate plot (**left** panel) and Breiman–Cutler variable importance plot (**right** panel) in random forest model.

The AUC value in training data was 0.991 (0.984–0.999 95% C.I.), and 0.865 (0.774–0.955 C.I.) in validation data. We found a slightly greater discrimination ability than that obtained with the logistic regression model in the validation data. Random forest is an additive model of classification trees where each model is built with different data and set of variables, to quantify the effect of the predictor variables to predict acidemia, we show in Figure 4 the variable importance (VIMP) plot. The VIMP measures the difference between prediction error under a perturbed predictor, where a permutation is designed to push a variable to a terminal node different than its original assignment, and the original predictor, these are calculated for each tree and averaged over the forest. This yields Breiman–Cutler VIMP [48]. The most influential variables in the prediction of acidemia were the number of decelerations with a deep greater than 60 beats per minute, the reperfusion time and the number of decelerations greater than 60 s.

Additionally, neural networks were trained with different architectures. We used the multilayer perceptron model with 1 or 2 hidden layers, different activation functions, initial weights, and training parameters. The best model on validation data was attained using the 13-10-1 architecture with 151 weights, and the activation function was logistic. The cross-entropy was used as the optimization function, this loss function measures the discrepancy between predictions and real occurrence of acidemia.

(1) $$E=-\frac{1}{N}\sum_{i=1}^{N}y_{i}\cdot \log\left(p\left(y_{i}\right)\right)+(1-y_{i})\cdot \log\left(1-p\left(y_{i}\right)\right)$$

being $y_{i}$ the dichotomic outcome, acidotic ($y_{i}$ = 1) or non-acidotic ($y_{i}$ = 0), and $p\left(y_{i}\right)$ the predicted probability of being acidotic for observation i out of N observations.

**Figure 5:**
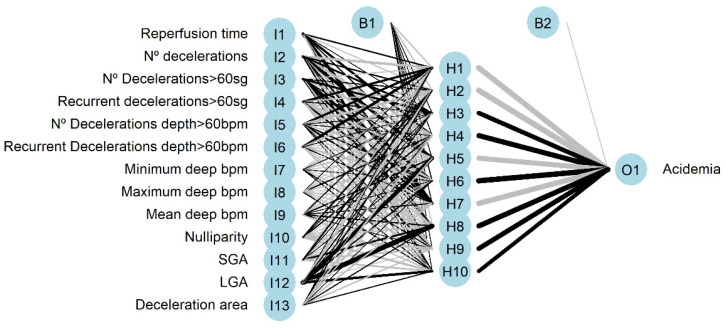
Neural network architecture with input (I), hidden (H), and output (O) layers. (B) is the result obtained after applying the activation function.

The architecture of the network is plotted in Figure 5, positive weights between layers are plotted as black lines, and the negative weights as grey lines. Line thickness is in proportion to relative magnitude of each weight.

**Figure 6:**
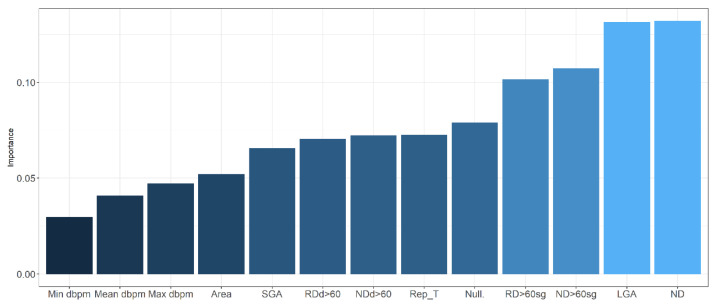
Variable importance in neural network. dbpm: deep in beats per minute; SGA: small for gestational age; RDd > 60: recurrent decelerations depth > 60 beats per minute; NDd > 60: number of decelerations depth > 60 beats per minute; Rep_T: reperfusion time; Null: nulliparity; RD > 60 sg: recurrent decelerations > 60 s; ND > 60 sg: number of decelerations > 60 s; LGA: large for gestational age; ND: number of decelerations.

The neural networks had an AUC value of 0.995 (0.985–1) (95% C.I.) for training data and 0.857 (0.751–0.963 95% C.I.) for validation data, greater than that obtained using logistic regression model but lower than the AUC that corresponds to random forest. Additionally, we present the variable importance plot for the multilayer perceptron, shown in Figure 6, following the method described by Garson 1991 [49], where the relative importance of explanatory variables for a single response in a supervised neural network is estimated by deconstructing the model weights. The most influential variables were the number of decelerations, being large for gestational age fetus, and the number of decelerations greater than 60 s.

#### 3.2.2. Validation of Models

**Figure 7:**
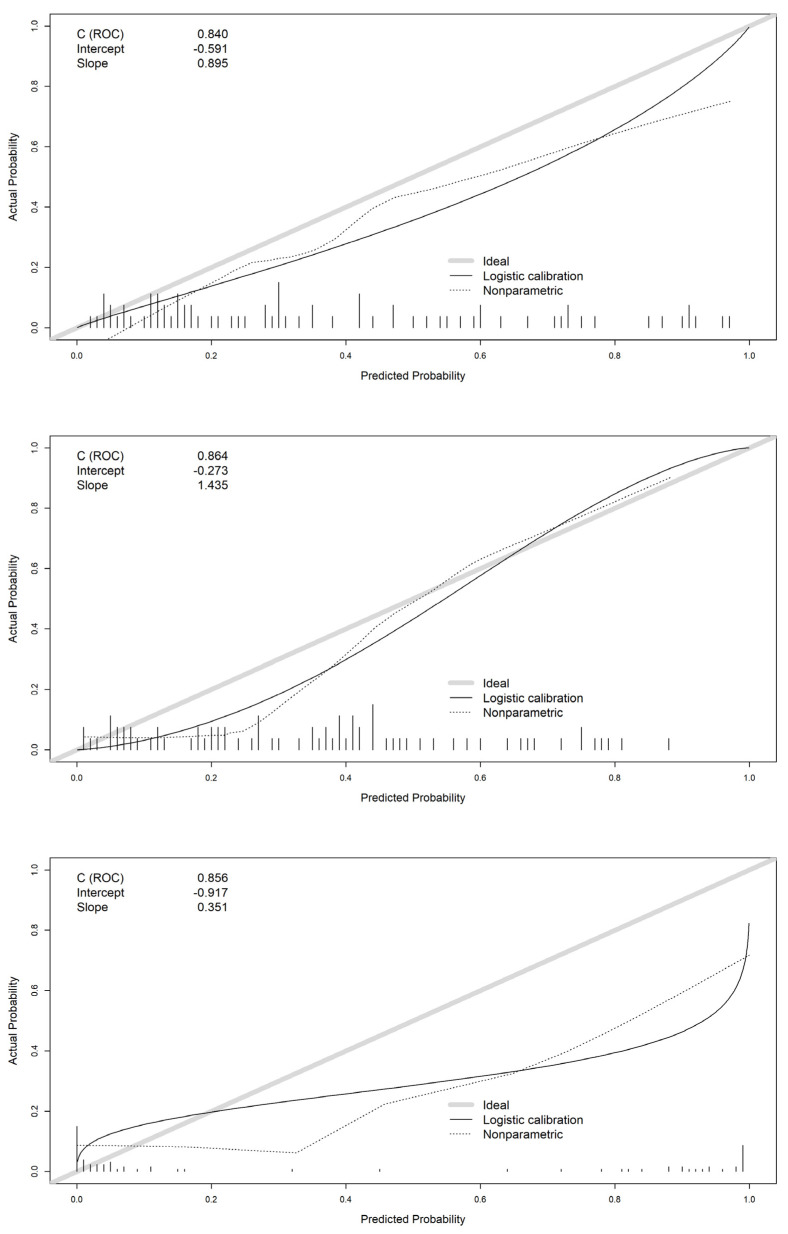
Calibration curves of logistic regression (**top** panel), random forest (**center** panel), and neural network (**bottom** panel) models.

In this section, we present the validation of the models developed using the validation data. The agreement between predictions and real outcomes was analyzed by calibration curves in Figure 7. For the logistic regression model, we found an overestimation of real acidemia occurrence, this is even more clear for neural networks. In the X axis of the graph, we show the predicted probabilities provided by models, for a 60% probability of acidemia, the actual occurrence of acidosis (Y axis) was 40% for logistic regression model, and 30% for neural networks, therefore, both models overestimate the real occurrence of acidosis.

For the random forest model, this overestimation was present only for probabilities below 0.4. The intercept showed also worse mean predictions for logistic regression (−0.591) and neural networks (−0.917) than random forest (−0.273) which is closer to 0. The slope was closer to 1 for logistic regression (0.895) with better concordance between predicted probabilities and real outcome.

**Figure 8:**
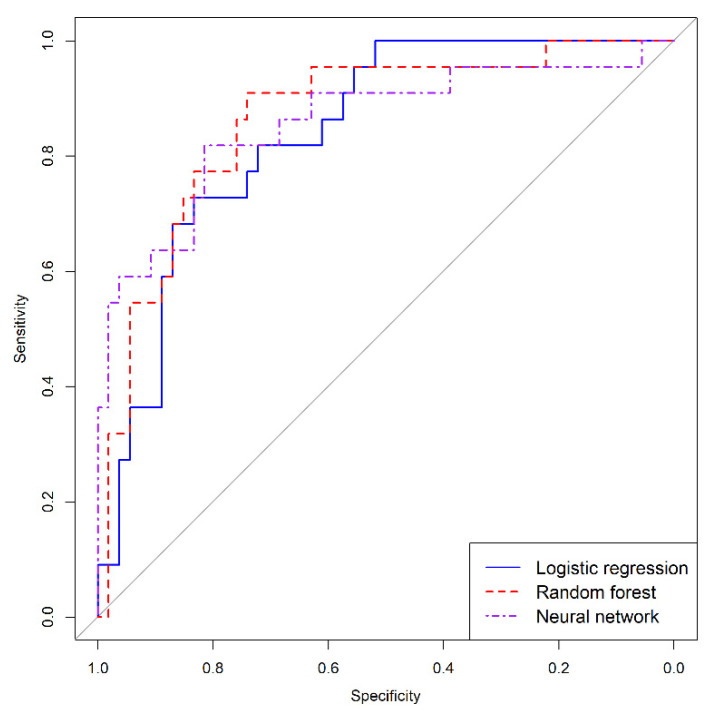
Receiver characteristic curves of logistic regression, random forest, and neural network models.

The discrimination ability of models is shown by ROC curves in Figure 8. All models show a good discrimination capacity. To compare the AUC of the models, we used the Delong comparison test. Differences between areas were not significant in our study, logistic regression vs. random forest (*p* = 0.561), logistic regression vs. neural networks (*p* = 0.736), random forest vs. neural networks (*p* = 0.888).

Finally, the clinical utility of models was analyzed. As our purpose was to predict acidemia, the most important issue was to analyze, for different threshold points, the false negative cases, that is, patients that by means of a cut-off point are going to be classified as non-acidotic below the cut-off point being acidotic. In the clinical utility curve, we analyzed this measure and the number of cases below a cut-off point, which in our study are candidates to a cesarean section that are going to avoid it.

**Figure 9:**
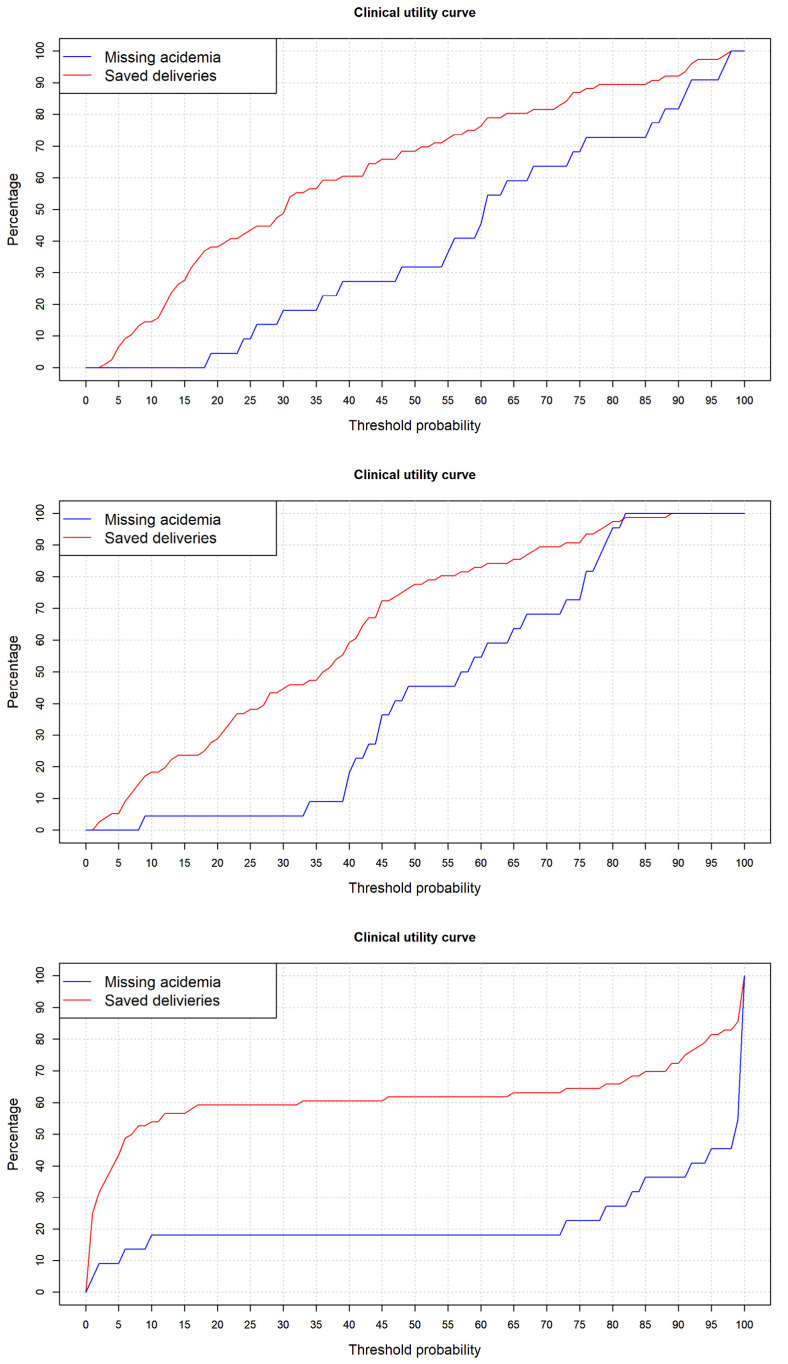
Clinical utility curve for logistic regression (**top** panel), random forest (**center** panel), and neural network (**bottom** panel) models.

Figure 9 presents the clinical utility curves. If we choose a maximum admissible level of 5% missing acidemia cases wrongly classified, in the curves we can analyze the threshold point that corresponds to this value. For the logistic regression model, this corresponds to a 23% cut-off point, and the number of deliveries saved with a minimum loss of acidotic cases was 40.8%. For the random forest model, it corresponds to a 33% acidotic probability threshold point, with 46.1% saved deliveries. Finally, for the neural network, it corresponds to a 1% cut-off point with 25% saved deliveries. Considering the clinical utility of the models, it is clear that the random forest proved superior.

## 4. Discussion

Here, we developed a comparison analysis of machine learning techniques to predict acidemia using FHR variables derived from the last 30 min of a continuous electronic fetal monitoring during intrapartum period. Built models showed a good and similar discrimination ability, but with clear differences in the calibration and clinical utility analysis, in which the random forest model showed the best performance.

The external monitoring of FHR is based mainly on the transmission of a transducer placed on the maternal abdomen, binding by an elastic band encircling the abdomen, localized at the fetal heart, although there is variability on CGT monitors [2]. Conductive gel placed between the abdomen and sensor favors the transmission of sound waves, but the signal can be affected by movement of maternal vessels or the fetus extremities, causing artefacts. This is a limitation for all systems that try to predict acidemia in real time, specially, in cases where the signal must be processed as in fractal analysis [17,18].

The development of devices to extract and monitor data should be followed by new software to analyze the FHR. The information theory is an essential issue to transmit, process, analyze data, and provide accurate information to the obstetrician in real time. In this context, there is a variety of applications of the theory of information in signal processing [50]. The digitalization of the signal provides the possibility of processing it by means of convolutional type networks or even more complex encoder–decoder deep learning structures in order to predict acidemia. Tang [27] designs a convolution neural network (CNN) model named MKNet with an AUC value of 0.95, they proposed their use by a real-time monitoring of fetal health on portable devices. Zhao [28] also uses CNN to provide predictions with an AUC above 0.95 in a 10-fold cross validation procedure. The accuracy of both models is very high but there is no analysis of calibration and clinical utility.

A different approach to the modeling of the complete signal is the extraction of variables from the signal that are combined in binary classification models of acidemia. In our analysis, we trained logistic regression, random forest, and neural networks using as predictor variables EFM features easily obtained from the EFM recording. Our best model was reached using random forest algorithm. These additive models provide robust models as their prediction is based on the sum of combination of trees building using different sets of data and variables. In our study, the best model was found using 100 trees, those trees are built using the 40% of predictor variables and 63% of the training data sample. The purpose of this selection is to guarantee that each tree explores the predictive ability of predictor variables in different data sample and over a different set of variables. In addition, the trees had a maximum number of cases at a terminal node of 5, preventing the overfitting that occurs in trees with too many branches.

The AUC obtained in validation data was 0.86, below results of the previous CNN models [27,28], but with good accuracy. Unfortunately, these studies lack a complete validation analysis, this would make them more comparable with ours. In our calibration analysis, we found that probabilities of acidemia provided by logistic regression and random forest model are well distributed in a wide range between 0 and 1. By contrast, in neural networks most probabilities are very close to 0 and 1, this is a clear sign of overfitting in the model. As a consequence, it is very difficult to choose a threshold probability point that separates acidotic and non-acidotic cases because probabilities are very concentrated in a narrow range. Logistic regression and random forest are more robust models, allowing the analysis of the advantages and disadvantages in terms of wrong classification of acidotic cases and avoided cesarean sections. In the case of the random forest model, to prevent 46% of unnecessary deliveries with a minimum loss of 5% of acidotic cases is a promising result.

Zhao [24] used an AdaBoost model with sensitivity of 92%, and specificity of 90%, similar to our results, showing the robustness of the additive tree models, although there is no information about how many cesarean sections could be saved with the 10% of academic cases wrongly classified. Iraji [29] used neural networks to reach a sensitivity of 99% and specificity of 97% which is near perfect classification. These values are extremely high and probably need an external validation to verify them. Balayla [20] in a metanalysis conclude that the use of AI and computer analysis for the interpretation of EFM during labor does not improve neonatal, but their conclusions are based only on risk ratio analysis. As we showed in our study, global measures of accuracy such as AUC can give the appearance that models are very similar, but their performance should be further explored using a complete validation process.

As a strength of our study, we found a classification model developed by means of a machine learning algorithm applied to EFM features that are easy to obtain from EFM recording. These predictor variables have proved as good predictors of acidemia in previous studies [14,15], but few studies have combined them in a predictive model using different machine learning algorithms. In addition, this model has shown good clinical utility to apply it in real clinical practice.

A limitation of the study is that it was a retrospective analysis with data sourced from a unique hospital without an external validation.

## 5. Conclusions

Using EFM recording, based on fetal resilience parameters, we developed a random forest model to predict acidemia that showed good accuracy, with AUC = 0.86 in validation data. This model can be applied in clinical practice using a cutoff point of 33% for the probability of acidemia, that showed 5% of missing acidemia but prevented 46% of unnecessary cesarean sections.

## Figures and Tables

**Table 1:** Descriptive characteristics.

Variable	Acidotic (*n* = 120)	Non-Acidotic (*n* = 258)	*p*-Value
**Maternal–fetal variables**			
Maternal age	33 (29–37)	34 (30–36)	0.499
Hypertension disorders	5 (4.2%)	5 (1.9%)	0.362
Gestational diabetes	15 (12.5)	29 (11.2%)	0.855
Nulliparity	132 (51.2%)	80 (66.7%)	0.007
Gestational age	280 (274–285)	280 (273–286)	0.841
Male gender	64 (53.3%)	145 (56.2%)	0.681
Delivery			<0.001
Vaginal	60 (50.0%)	187 (72.5%)	
Operative vaginal	30 (25.0%)	52 (20.1%)	
Cesarean	30 (25.0%)	19 (7.4%)	
Birthweight	3238 (2918–3638)	3295 (2975–3620)	0.645
Percentile birthweight	43. 1 (20.0–74.5)	49.3 (24.1–77.4)	0.553
Small for gestational age	22 (18.3%)	28 (10.9%)	0.066
Large for gestational age	21 (17.5%)	32 (12.4%)	0.185
**EFM variables**			
ACOG categories			<0.001
Category 1	13 (10.8%)	123 (47.7%)	
Category 2	57 (47.5%)	110 (42.6%)	
Category 3	50 (41.7%)	25 (9.7%)	
Reperfusion time (min)	18.1 (14.8–20.8)	21.8 (18.2–25.2)	<0.001
Number of decelerations	8 (5–10)	7.5 (4–10)	0.509
№ Decelerations > 60 sg	2.5 (0–5)	0 (0–2)	<0.001
Recurrent decelerations > 60 sg	25 (20.8%)	20 (7.8%)	<0.001
№ Decelerations depth > 60 bpm	3 (1–5)	0 (0–3)	<0.001
Recurrent decelerations depth > 60 bpm	33 (27.5%)	43 (16.7%)	0.021
Deceleration area	16.5 (11.3–22.6)	9.6 (5.1–15.5)	<0.001
Minimum deep bpm	40 (30–54)	31 (24–40)	<0.001
Maximum deep bpm	79 (68–92)	60 (52–78)	<0.001
Mean deep bpm	58 (48–69)	48 (40–69)	<0.001

EFM: electro fetal monitoring; ACOG: American College of Obstetricians and Gynecologists; bpm: beats per minute.

**Table 2:** Multivariate logistic regression model.

Variable	Odds Ratio (95% C.I.)	*p*-Value
Nulliparity	0.413 (0.217–0.763)	0.006
Large for gestational age	4.562 (1.969–10.840)	<0.001
Reperfusion time (min)	0.809 (0.729–0.889)	<0.001
Number of decelerations	0.804 (0.694–0.919)	0.002
№ Decelerations > 60 sg	1.190 (1.037–1.369)	0.013
№ Decelerations depth > 60 bpm	1.328 (1.111–1.599)	0.002
Recurrent decelerations depth > 60 bpm	0.178 (0.056–0.530)	0.005
Minimum deep bpm	1.034 (1.010–1.060)	<0.001

**Table 3:** Random forest parameter configuration.

Parameter	Value
Number of trees	100
Forest terminal node size	5
Average number of terminal nodes	29.9
Resampling used to grow trees	SWOR
Resample size used to grow trees	191
Splitting rule	MSE
Number of random split points	10

SWOR: sampling without replacement; MSE: mean squared error.

## Data Availability

The data analyzed were retrieved from the Miguel Servet University Hospital database.
